# Treating Lows: Management of Orthostatic Hypotension

**DOI:** 10.1097/FJC.0000000000001597

**Published:** 2024-09-03

**Authors:** Spoorthy Kulkarni, Danny Jenkins, Arko Dhar, Fraz Mir

**Affiliations:** * Department of Medicine, University of Cambridge, Cambridge, United Kingdom;; † Department of Clinical Pharmacology and Therapeutics, Cambridge University Hospitals NHS Foundation Trust, Cambridge, United Kingdom;; ‡ Lewisham and Greenwich NHS Foundation Trust, London, United Kingdom; and; § University of Mississippi Medical Center, Jackson, Mississippi, United States of America.

**Keywords:** orthostatic hypotension, hypertension, pharmacology, syncope

## Abstract

Orthostatic hypotension is a prevalent clinical condition, caused by heterogenous etiologies and associated with significant morbidity and mortality. Management is particularly challenging in patients with uncontrolled hypertension. A thorough assessment is needed to draw an appropriate management plan. The treatment aims to improve postural symptoms while minimizing side effects and reducing iatrogenic exacerbation of supine hypertension. A personalized management plan including rationalizing medications, patient education, identification, and avoidance of triggers, as well as nonpharmacological therapies such as compression devices, dietary modifications, and postural aids, make the first steps. Among pharmacological therapies, midodrine and fludrocortisone are the most prescribed and best studied; pyridostigmine, atomoxetine, and droxidopa are considered next. Yohimbine remains an investigational agent. A multidisciplinary team may be required in some patients with multiple comorbidities and polypharmacy. However, there is a lack of robust efficacy and safety evidence for all therapies. Building robust real-world and stratified clinical trials based on underlying pathophysiology may pave the way for further drug development and better clinical strategies and in this challenging unmet medical need.

## Abstract 2

Supplemental Digital Content is Available in the Text.

## INTRODUCTION

The ability to stand and engage in activities while remaining standing is one of the fundamental characteristics that differentiates human beings from many other species. This maneuver uses intricately programmed complex physiological processes. Postural or orthostatic hypotension (OH) results from an inadequate physiological response in blood pressure (BP) to postural changes and is typically defined as a sustained drop in systolic BP (SBP) of ≥20 mm Hg and/or diastolic BP (DBP) of ≥10 mm Hg within the first 3 minutes of standing from a supine position or of tilting the body (head up) to at least a 60-degree angle on a tilt table.^1,2^ A reduction in SBP of ≥30 mm Hg is considered significant in patients with a diagnosis of hypertension.^3^ OH tends to be deemed as being clinically significant when accompanied by the classical symptoms or a presentation of “dizziness” or “falls.”

Prevalence of OH varies from 6% to 55% in the general adult population, expectedly increasing with age.^4–6^ OH has been associated with an increased risk of ischemic stroke, coronary events, heart failure, and all-cause mortality,^7^ especially in specific patient cohorts with underlying diabetes mellitus (DM).^8^ Aging, concomitant medications [commonly antihypertensive including diuretics and vasodilators including drugs for benign prostatic hyperplasia (BPH)], smoking, low body mass index, hypertension, and DM are known risk factors for OH. OH is a common early feature in Parkinson's disease (PD) and dementia with Lewy bodies, with OH preceding the diagnosis of these neurological conditions by up to 4 years.^9^ Approximately 10%–25% of patients with DM (type 1 and type 2) and 16%–58% of patients with PD are affected by OH.^10,11^ Poor glycemic control as indicated by glycated hemoglobin is a key factor in the development of OH in type 2 DM patients and this relationship is particularly pronounced in elderly patients,^12^ hypertension, and insulin treatment.^13^ OH is also commonly associated with acute systemic illness including direct infection,^14,15^ involving the autonomic nervous system (ANS),^16^ or an indirect effect, eg, dehydration and associated deconditioning associated with sepsis and recovery. Asymptomatic and incidental OH is common in hospital inpatients and the management is usually dependent on the impact of OH on the patient. The prevalence of OH in acute geriatric wards was estimated to be 67.9% (mean age of 81.6 years), in a study where OH was measured as a drop in BP at least once per day.^17^ A period of illness, disturbances in electrolyte and water homeostasis, and in-hospital stay, especially in the elderly, can contribute to physical deconditioning, triggering the development of OH. Other infections, for instance, Lyme disease and COVID-19, have been associated with acute and postinfectious persistent autonomic disturbances in patients.

One of the most frequently associated noninfectious comorbidities with OH is hypertension; approximately 8.9% of patients with OH have a diagnosis of essential hypertension,^18^ which also then poses a therapeutic challenge. OH can be particularly problematic in patients with chronic kidney disease treated with dialysis because of rapid fluid shifts and made worse by associated hypertension and anemia. Rarely, subacute OH may be a manifestation of paraneoplastic autoimmune syndromes in patients with lung cancer and multiple myeloma. The exact prevalence of OH in elderly patients with other comorbidities and associated polypharmacy for conditions such as BPH is unknown.

Some patients can tolerate large drops in BP without any symptoms, whereas others develop symptoms with a minimal drop, regardless of whether the standing BP is within the “normal range.” Most patients tend to develop symptoms when the BP drops below the lower limit of the cerebral autoregulatory range, which in most patients ranges from 50 to 150 mm Hg of mean arterial pressure. Identifying asymptomatic OH may be helpful in some patients, though the true prevalence rate is unknown and whether treating asymptomatic OH improves morbidity or mortality is unclear.

The high prevalence of OH and comorbidities associated with it underscores the importance of identifying, assessing appropriately, and drawing up individualized management plans for patients. In this review, we aim to bring to outline the pathophysiology, explore the potential causes of OH based on presentations, and present a comprehensive approach to diagnosing and managing these patients based on current and plausible treatment strategies based on the evidence available.

## METHODS

To review the evidence base for the currently used and newer therapies in drug development of OH, PubMed and Embase databases were searched, using search terms including “OH” or “postural hypotension” (PH), “therapy” and “clinical studies” or “randomized controlled trials” and its synonyms. All studies that at least had ≥10 participants were included in the evidence summary. Further relevant studies were hand-searched based on references.

## PATHOPHYSIOLOGY, CLASSIFICATION, AND ETIOLOGY OF OH

At its core, OH is a failure of the normal autonomic response of the body to postural changes. BP homeostasis is regulated by global and local responses. Central mechanisms rely on cardiac output (CO) and total peripheral resistance (TPR) to ensure an adequate BP for tissue perfusion. There are 3 key sets of “pressure sensors”: high-pressure baroreceptors (carotid sinus, aortic arch, and afferent renal arterioles), carotid and aortic body chemoreceptors, and cardiopulmonary baroreceptors.

**FIGURE 1.**
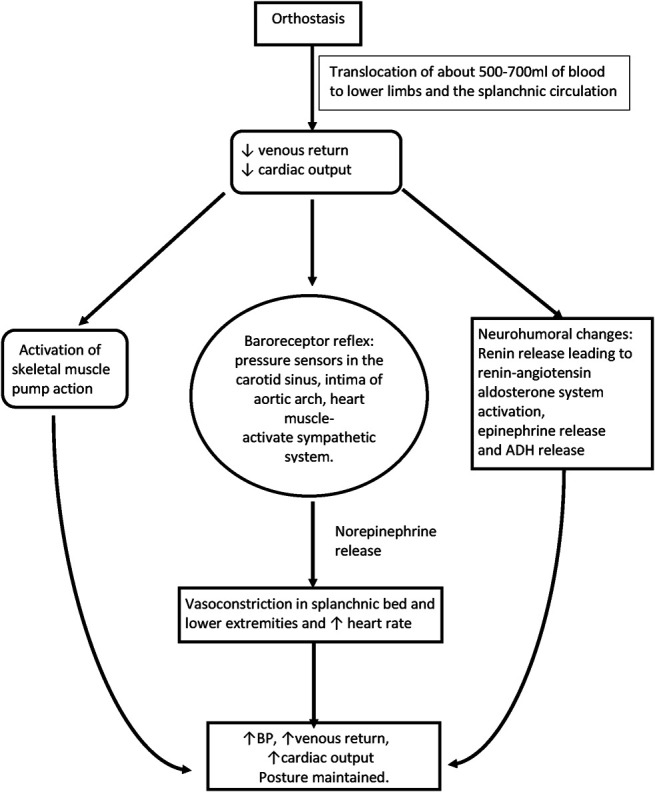
Physiological compensatory mechanisms that maintain blood pressure and heart rate on standing. ADH, antidiuretic hormone; BP, blood pressure.

The act of standing up results in the translocation of approximately 500–700 mL of blood to the peripheral venous capacitance vessels, mainly in the lower limbs and the splanchnic circulation by gravity. This in turn leads to a decrease in venous return and a reduction in CO by approximately 20%. These changes are normally counteracted by the disinhibition of sympathetic efferent signals from the cardiovascular (CV) center in the medulla. The actions of the sympathetic efferent nerves increase CO not only by uninhibited sympathetic stimulation of cardiac tissue but majorly by the vasoconstriction of lower limb skeletal muscle and gastrointestinal precapillary blood vessels that raise the TPR and the mean systemic filling pressure in the presence of an intact baroreflex. Combined, these actions ensure that arterial BP is maintained on standing up from the supine position as depicted in Figure 1. Although the baroreflex is a very short-acting feedback loop, activated within seconds, the renin–angiotensin–aldosterone system (RAAS) can take minutes to hours to be activated. Standing up results in the production of renin because of a transient reduction in afferent arteriole perfusion pressure, which in turn activates angiotensin II and aldosterone production. Angiotensin II itself causes vasoconstriction and acts centrally to increase sympathetic outflow. OH can result from the failure of activation of any or all these pathways.

**FIGURE 2.**
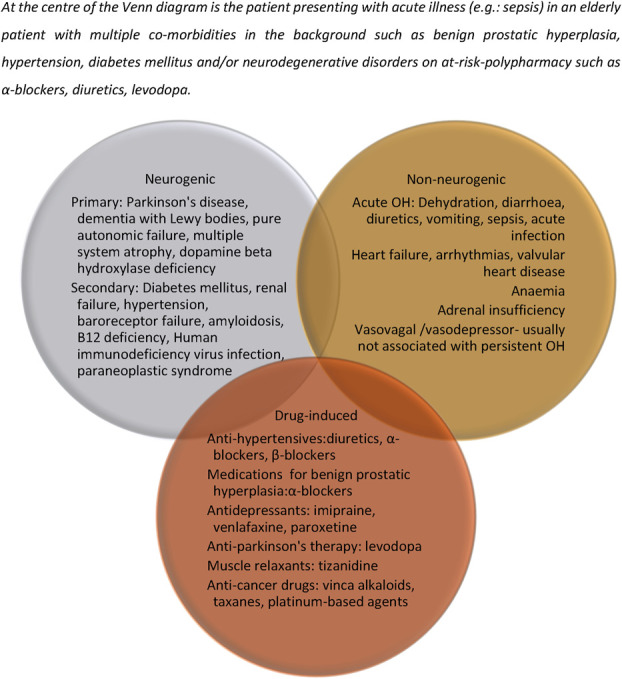
Conditions associated with orthostatic hypotension. At the centre of the Venn diagram is the patient presenting with acute illness (e.g., sepsis) in an elderly patient with multiple co-morbidities in the background such as benign prostatic hyperplasia, hypertension, diabetes mellitus, and/or neurodegenerative disorders on at-risk-polypharmacy such as α-blockers, diuretics, and levodopa.

The major etiologies that are associated with OH are listed in Figure 2.^5,19,20^ Any dysfunction of the ANS resulting in inadequate release of norepinephrine from efferent sympathetic vasomotor nerves leads to vasoconstrictor failure, and the resultant OH is identified as neurogenic OH (nOH). nOH can be further subdivided into primary, characterized by primary degeneration of the autonomic nerves, and secondary (systemic etiology affecting peripheral nerves). PD, pure autonomic failure (PAF), dementia with Lewy bodies, and multisystem atrophy (MSA) are the most common causes of primary nOH.^6^ Furthermore, in MSA, the central autonomic neurons are affected, and the peripheral neurons are spared, in contrast to other conditions, where the pattern of involvement is reversed. This distinction can have important treatment considerations. DM, alcoholic polyneuropathy, amyloidosis, and paraneoplastic syndromes affecting peripheral nerves are examples of secondary nOH. Non-nOH usually refers to OH in the light of fully preserved ANS, indicating an underlying reduction in circulating blood volume or conditions affecting the pumping action of the heart. Dehydration, drugs, age, heart failure, and acute illnesses are the usual causes.

Elderly patients are particularly prone to OH because of the presence of multiple etiological factors. For example, BPH is prevalent in elderly men as is DM and hypertension. Usual pharmacological treatment options for BPH include α1-adrenergic antagonists. There are 3 types of α1-adrenergic receptors α-1A, α-1B, and α-1D. Tamsulosin and alfuzosin are licensed in the United Kingdom for BPH because they reduce smooth muscle tone of the bladder neck (using α-1A receptors) thereby improving urine flow. Nonspecific α1 blockers such as doxazosin, prazosin, and terazosin are relatively nonspecific α1-adrenergic receptors and lead to significant vasodilatation and are generally preferred for BPH and concomitant hypertension. However, in vitro studies show that alfuzosin is nonselective and tamsulosin is equipotent at α-1A and α-1D and with a higher selectivity (35-fold) α-1A versus α-1B,^21^ and thereby, patients who are prescribed these medications may still be at risk of OH and hypotension. This study also demonstrated high affinities of some of commonly prescribed the anti-depressants (tricyclic anti-depressants) and anti-psychotics (first and second generation) to cause α-blockade.

There are subtle differences between presentations of OH based on pathophysiology that can help delineate the underlying etiology and streamline therapeutic options. Concomitant measurement of heart rate (HR) when testing for OH provides crucial information, which helps to differentiate between the 2 major categories. Non-nOH is typically associated with a compensatory increase in HR (∼15 beats per minute). Patients with nOH tend to present with more profound drops in BP, usually persistent with every orthostatic challenge. Patients with nOH may also develop adaptive changes in cerebral autoregulatory mechanisms and hence may be able to tolerate wider swings in BP and remain asymptomatic at significantly lower BP values. Conversely, an immediate asymptomatic drop in BP within 5–15 seconds of standing, which resolves quickly (by 20 seconds), is sometimes observed in healthy adults^22^ and generally does not need pharmacotherapy. Supine hypertension (SH) is a frequent accompanying feature, especially in patients with nOH. Postprandial hypotension (PPH), ie, a reduction in SBP of ≥20 mm Hg within 2 hours of a meal, is commonly associated with nOH, resulting from the dilatation of the splanchnic vasculature and the consequent loss of physiological reserve to maintain BP on standing. Gastrointestinal vasodilatory peptides and insulin release after meal ingestion are considered causally related to PPH.^23^

The differential diagnoses for patients experiencing symptoms of the orthostatic challenge include reflex vasovagal syncope and postural orthostatic tachycardia syndrome, which together can be grouped into an “orthostatic intolerance (OI) syndrome.” There is usually no significant fall in BP on standing in these patients on routine testing. However, patients prone to vasovagal syncope may have delayed OH and as such may be considered part of the spectrum. Vasovagal syncope is a complex set of events, characterized by paroxysmal withdrawal of sympathetic vasopressor tone, often triggered by prolonged standing, resulting in a fall in BP, HR, and subsequent transient loss of consciousness secondary to parasympathetic stimulation.

### OH in Hypertensive Patients

Elderly patients with hypertension may be prone to OH because age-related vascular stiffening can lead to decreased baroreceptor sensitivity (afferent part of the reflex)^24^ presenting as a particularly challenging therapeutic conundrum.

Most antihypertensive therapies make OH worse in the short term, and some classes of medication are worse than others (see under assessment section). As cerebral autoregulation is shifted to the right in patients with hypertension, patients may not tolerate standing BP even if in the normal range in comparison with patients without hypertension. Juraschek et al explored whether aggressive antihypertensive treatment can prevent the development of OH in an individual participant meta-analysis of RCTs. Indeed, the odds of developing OH were lower in the intensive treatment group relative to the standard treatment group with a low degree of heterogeneity.^25^ This finding was observed in groups including adults without DM and adults with standing SBP below 110 mm Hg, a group not assessed in prior OH reviews. This result was likewise found in adults with a seated SBP of 140 mm Hg or greater, DBP of 90 mm Hg or greater, history of stroke, eFGR below 60 mL/min per 1.73 m^2^, obesity status, and history of CV disease.^25^ Of note, the included studies did not consider major sequelae of OH including syncope and falls among patients as an outcome, and the strict inclusion criteria for the different studies also meant that more frail patient populations were excluded. As such, the patients in the clinic may not be representative of the included patients in that review. Nonetheless, the evidence seems to favor the treatment of hypertension in patients with OH rather than not. The rationale for doing so reflects the physiological mechanisms at play—those with significant SH while asleep, for instance, experience a compensatory pressure natriuresis that in turn exacerbates their OH during the day. Treating SH, eg, using short-acting formulations of calcium blockers or transdermal nitrate patches minimizes the pressure natriuresis that subsequently attenuates BP drop on standing.^26^

### CLINICAL ASSESSMENT AND EVALUATION OF A PATIENT WITH OH

Symptoms that come on when assuming an upright position, sometimes on sitting, and improve upon lying down or sitting are a general prerequisite for diagnosis. Common symptoms include light-headedness or feeling faint, “dizziness”, blurred vision, fatigue, and occasionally syncope. Unusual yet intriguing symptoms include discomfort in the neck and shoulders (referred to as “coat hanger pain” because of hypoperfusion of the neck muscles), shortness of breath (ventilation/perfusion mismatch in the apical lung areas), and generalized weakness or fatigue. OH should be particularly excluded in patients with hypertension and patients reporting recurrent falls and syncope.

Most patients experience OH in the morning. This diurnal variation is because of a reversed pattern of natriuresis, usually associated with a reversed BP diurnal variation, resulting in significant nocturia, and reduced intravascular volume at night, the effects of which are seen when the patient wakes up in the morning.^27^ In patients with non-nOH, particularly with vasovagal presyncope, symptoms of autonomic activation including diaphoresis, nausea, abdominal pain, or diarrhea may be present, in contrast to patients with nOH, where they are often conspicuously absent.

**FIGURE 3.**
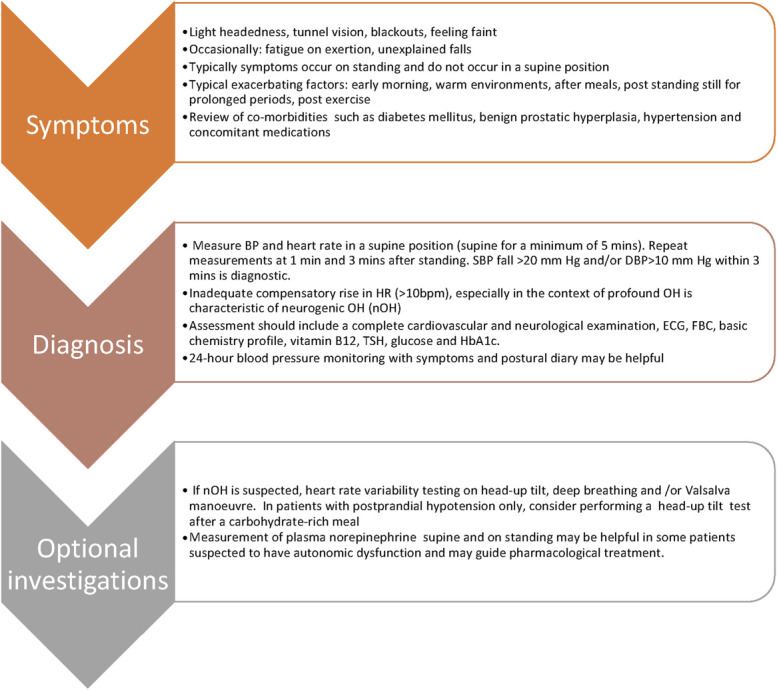
Clinical presentation and evaluation of patients with suspected OH. BP, blood pressure; ECG, electrocardiogram; FBC, full blood count; TSH, thyroid-stimulating hormone.

A detailed history including medication review, a thorough focused examination and an assessment of the impact of OH is a prerequisite to determining the factors that are contributing to physiological derangement (Fig. 3). Modifiable exacerbating factors that cause vasodilatation such as exercise, heat, or dehydration should be identified and addressed. Numerous commonly prescribed pharmacotherapies can result in OH as an on/off-target effect, tricyclic antidepressants, diuretics, anti-PD therapies, and vasodilators to name a few (Fig. 2). Among antihypertensive therapies, there is a suggestion that nitrates, α-blockers, β-blockers, and diuretics tend to worsen OH more in comparison with RAAS inhibitors and calcium channel blockers.^28^

The first and foremost step is to measure lying and standing BP along with HR (Fig. 3). The patient should be resting for 5 minutes before standing up. A cross-sectional study comparing sit-to-stand maneuver to lying-to-standing BP showed optimal sensitivity and specificity with lower diagnostic cut-offs, namely, a drop of 15 mm Hg for SBP and 7 mm Hg for DBP.^29^ Repetition of testing is important in a patient who has typical symptoms of OH but with negative findings on initial assessment, occasionally including monitoring BP and HR at home or with 24-hour ambulatory BP monitoring along with a symptom and activity diary can be revealing. The latter could also detect SH and PPH and guide the doses and timing of pharmacotherapy.

Apart from lying and standing, BP values, routine biochemistry, and hematology to look for conditions such as undiagnosed DM, hypoadrenalism, renal failure, or anemia ought to be undertaken, depending on the corroborative history. A complete assessment of the neurological and CV systems, including electrocardiogram, is essential in all cases, and prolonged electrocardiogram monitoring and echocardiogram (particularly in the presence of concomitant CV comorbidities) may be recommended in some patients if cardiac causes such as aortic stenosis and cardiomyopathies are suspected. Geriatric assessment in elderly patient cohorts is crucial for designing long-term management plans including a “falls” prevention plan. Various methods exist to detect failure of vaso-vagal reflex such as measurement of TPR in response to orthostasis. Beat-to-beat HR variability (BP and CO) on deep breathing and/or response to Valsalva maneuver are usually abnormal in nOH reflecting baroreflex–cardiovagal failure; hence, assessing this may be useful in some cases especially in nOH. Lying and standing plasma norepinephrine level measurements may be useful in selected patients with nOH. Normally 100% increase in plasma norepinephrine after 5–10 minutes of standing would be expected, and an increase by <60% may suggest nOH. Typically, in MSA, baseline norepinephrine levels are near normal, whereas in PAF, levels are lower. In PD, levels may be low, but levels may be normal; however, that does not necessarily indicate normal sympathetic innervation. Categorizing and assessing the sympathetic reserve may help guide and rationalize treatment.

## MANAGEMENT

The first step before prescribing any new medications is to rationalize pre-existing drugs, considerations focused on whether they can be stopped or adjusted (dose, formulation, or timing). α-1 Adrenergic receptor antagonists are one class of drugs that should be carefully assessed in the setting of OH because of their tendency to precipitate the condition. For example, if tamsulosin is required for benign prostatic hypertrophy, the time of intake can be changed to the nightly dose instead of the morning dose. Alternatively, it may be reasonable to switch it to a 5-α reductase inhibitor, eg, finasteride to “shrink” the prostate (over months) and therefore possibly obviate the need for an α-blocker. In patients with co-existent SH, night-time administration of a short-acting antihypertensive agent such as a nitrate patch or short-acting oral medication might be considered to combat risks associated with uncontrolled SH^30^ with caution.^26^

The goals of treatment are 2-fold, to halt the drop in standing BP and improve symptoms resulting in improvement in functionality. An ideal treatment strategy would have orthostatic pressor selectivity, ie, an increase in standing BP while having minimal effect on supine BP. To enable such a pharmacodynamic effect, quick onset of action and relatively short duration of action would be additional desirable properties. However, very few therapies currently available meet such a fit.

In clinical practice, ensuring that patients develop a basic understanding of OH to avoid exacerbating factors such as dehydration is the first vital step.

**FIGURE 4.**
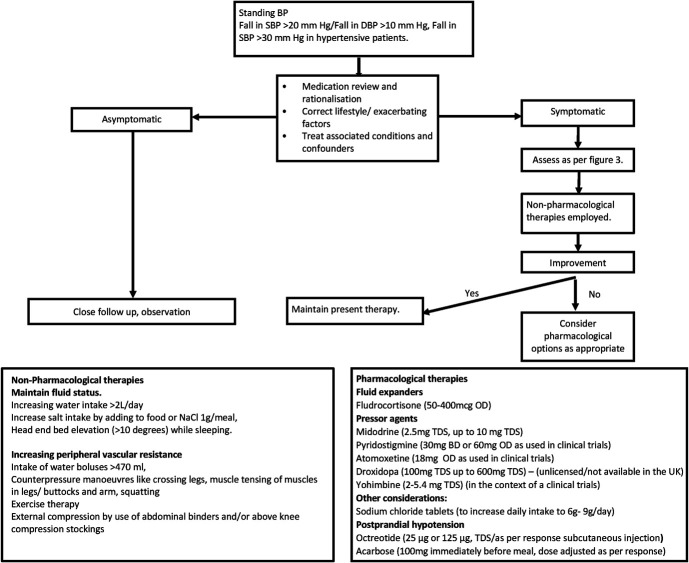
Proposed management algorithm including a summary of nonpharmacological and pharmacological therapies. TDS, three times daily.

Educating patients about their condition and empowering them to actively participate, which may include providing resources, access to support groups, occupational therapies and importantly clear instructions on managing symptoms, can significantly improve outcomes. Education ultimately motivates patients to identify, use, and persist with nonpharmacological and pharmacological therapies that benefit them most (Fig. 4). A multidisciplinary approach involving primary care physicians, cardiologists, neurologists, clinical pharmacologists, and other specialists may be necessary to provide comprehensive care for some patients with OH.

All therapeutic recommendations for OH are currently based on results from small studies undertaken in nOH, namely PD, PAF, and MSA The heterogeneity in underlying etiology leading to OH, lack of long-term data on efficacy, tolerability, and sustainability of using a particular intervention, patient factors including adherence to therapy and poor correlation of BP values with symptom improvement are some of the factors that make interpretation and pooling of outcomes of studies of nonpharmacological and pharmacological therapies challenging (see **Supplementary Table**, **Supplemental Digital Content 1**, http://links.lww.com/JCVP/B126).

### Assessment of Interventions or Therapy

The clinically used measure for therapy effectiveness for OH is the improvement in standing SBP at 1–3 minutes. Other commonly used surrogates include changes in ambulatory BP, head-up tilt test, and symptom improvement questionnaires; in particular, the Orthostatic Hypotension Questionnaire (OHQ) that can represent the patient-reported outcome measure (PROM). The Orthostatic Hypotension Questionnaire, the most used PROM in clinical research focused on OH, has 2 components, a 6-question symptom assessment (“dizziness, light-headedness, feeling faint, or feeling like you might black out”) and a 4-item daily activity scale.^31^ The Clinical Global Impressions scale is another PROM, which consists of 2 companion measures, the first evaluates the severity of psychopathology from 1 to 7 and the second assesses the change from the initiation of treatment on a similar 7-point scale.^32^ However, the applicability of these PROMs in clinical practice is not well-established.^33^ Likewise, PROM assessments in clinical practice could provide real-world robust comparisons of therapeutic benefits among the ones trialed, providing a benchmark for the most effective therapy specific for every patient.

### Nonpharmacological Therapies

Nonpharmacological therapies are the first strategies used in a patient with OH.

Exercise therapy, essentially strength and resistance training, has been postulated to lead to better adaptation to orthostatic challenges in both OH and OI syndromes.^34,35^ Swimming, cycling, and rowing are ideal exercises, usually performed in sitting or recumbent positions and have a positive impact on CV health overall too. However, exercise therapy, when studied in trials in the form of home-based resistance training, does not necessarily lead to sustained improvement in OH, especially in elderly patients.^36^

Physical maneuvers that reduce venous pooling of blood can increase CO by 1.3–1.7 times. These include crossed leg standing, leg muscle tensing, crash positioning, squatting, isometric arm exercises, and buttock clenching. They play a role in the prevention of syncopal events,^37,38^ with squatting showing the most consistent effect.^39,40^ When patients with a high risk of falls use these basic physical counterpressure maneuvers, the risk of syncopal falls can be reduced by 39%.^41^ However, their employability in elderly patients with OH is limited by dependence on muscular physiological reserve and as such nonfrail patients are likely to benefit.

Elevation of the bed head during sleep, typically to 12–30 degrees (or approximately 6–9 inches), keeps the RAAS axis primed and may prevent nocturnal natriuresis, thus blunting OH upon waking,^19,42–44^ although efficacy as such is ambiguous.^45^ Aside from tolerability issues, it is a worthwhile simple strategy for all patient cohorts. During the day, patients are mostly advised to avoid the supine position, especially if taking concomitant therapies such as fludrocortisone and midodrine.

Water boluses and supplementary sodium intake have been postulated to reduce OH by increasing the circulating volume. Oral water boluses activate a sympathetic pressor reflex, increasing BP, even in the face of baroreceptor failure.^46^ Increased sodium intake augments the circulating volume, partly by driving thirst. Water boluses of typically half a liter may be effective at attenuating an orthostatic drop in BP at 30 minutes post bolus by approximately 12 mm Hg.^47–50^ Although standing SBP improves, few studies demonstrate a reduction of the symptomatic burden.^51^

Sodium supplementation (6–10*g*/day) has been thought to improve OH by aiding intravascular volume expansion,^50^ although little evidence exists to support this. For the elderly patient at the center of the Venn diagram in Figure 2, with devastating OH along with SH and heart failure, sodium supplementation is best avoided. No adverse effects are noted in younger cohorts, although caution must be exercised in the regular use of salt supplementation for OH treatment as a stand-alone therapy.

Compression stockings and body binders (lower limbs and abdomen) reduce the fall in BP compared with sham compression in patients with non-nOH, relying on external compression to improve venous return^52^ with a sustained impact noticeable for up to 4 weeks. Compression stockings seem to have modest effects.^53–57^ Similarly, abdominal binders work by reducing splanchnic venous pooling under orthostatic stress. They seem to be more effective than the compression of other capacitance beds (calf and thighs) with an average rise in standing BP ranging from 10 to 12 mm Hg.^53,54,58^ Abdominal binders can be envisaged as an ideal treatment for OH because their effect on BP can be posture-selective and thus safer than other approaches.^59,60^ The main drawback of both abdominal binder and compression strategies is their long-term tolerability in patients. In very elderly and frail patients with cognitive dysfunction, their application can prove to be particularly challenging.

Small studies in patients with nOH have shown significant OH occurring after larger, less frequent meals.^61^ Smaller, more frequent meals, as opposed to large single meals, can reduce the frequency of PPH. Reduction in meal size may be a valid treatment option for PPH, but there is inadequate evidence to support it as a primary form of treatment. Likewise, a bedtime snack may be preferable in patients with co-existent SH because BP could lower as a response to eating, unless the patient has significant symptomatic nocturia and tends to stand up frequently at night in which case, it is imaginably detrimental.

Beyond traditional nonpharmacologic interventions for OH, a novel mechanism of external neurostimulation of preganglionic sympathetic nerves has been trialed. In a case report involving a 48-year-old woman suffering from nOH secondary to MSA, a neurostimulation device was implanted epiduraly at the level of the thoracic spinal cord.^62^ Gradually, after weeks of rehabilitation with the device, the patient had a marked decrease in syncopal episodes and was able to walk independently for greater distances. These experimental therapies warrant further investigation.

Overall, among nonpharmacological options, the use of abdominal binders, elevation of the bed head, and water boluses seem to have better convincing evidence for improvement in OH (BP and symptom improvement). In practice, counterpressure maneuvers do help prevent syncopal attacks to a certain degree. Regular, individualized exercise therapy plans may certainly help prevent further OH episodes in certain patients who can tolerate and maintain them.

### Pharmacological Therapies

**FIGURE 5.**
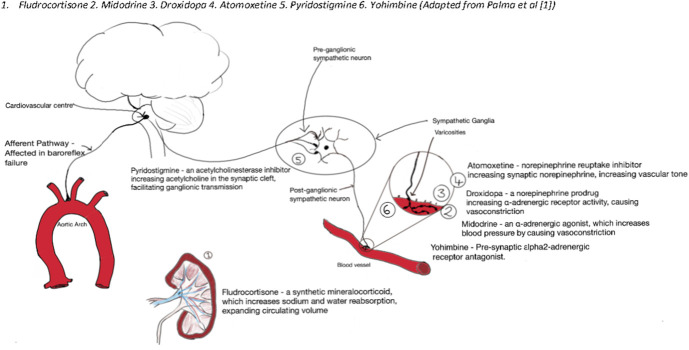
Site of action of pharmacotherapies used in orthostatic hypotension. 1. Fludrocortisone, 2. Midodrine, 3. Droxidopa, 4. Atomoxetine, 5. Pyridostigmine, 6. Yohimbine (Adapted from Palma et al^1^).

At present, there is no single effective drug of choice that can be claimed to be the pharmacological cure for OH, and their efficacy in individual patients seems to be highly variable. Instead, medications have been repurposed and/or used off-label for the treatment of OH, as summarized in Figure 4. The site of action of the most plausible pharmacological therapies has been illustrated in Figure 5. Table 1 provides a summary of characteristics of the most commonly used and studied therapeutics options for OH.

**TABLE 1.** Characteristics of Medications Tried for OH

Medication	Mechanism of Action	Dose Range	Contraindications	Side Effects
Atomoxetine	A selective norepinephrine reuptake inhibitor, increasing norepinephrine available in the synapse	18 mg used in trials (OD) Not licensed for OH in United Kingdom	Phaeochromocytoma; severe cardiovascular disease; and severe cerebrovascular disease	Anxiety: appetite decreased; asthenia; chills (in adults); constipation; depression; dizziness; drowsiness; gastrointestinal discomfort; headaches; hyperhidrosis; menstrual cycle irregularities; altered mood); nausea); prostatitis; sexual dysfunction; thirst; vomiting; and weight loss
Caffeine	Wide ranging and poorly understood mechanism—thought to stimulate catecholamine release causing vasoconstriction (when used irregularly)	100 mg used (OD)	No absolute CI, caution advised with history of severe anxiety; cardiovascular disease or symptomatic arrhythmias; peptic ulcer disease or gastroesophageal reflux disease	Restlessness and shakiness, insomnia, headaches, dizziness, tachycardia, dehydration, and anxiety
Droxidopa (L-DOPS)	A norepinephrine precursor, which is metabolised in the brain, allowing the norepinephrine to act on α-adrenergic receptors causing vasoconstriction and on beta-adrenergic receptors to stimulate heart contraction and dilate arteries	100–600 mg (TDS) Not available in United Kingdom	Neuroleptic malignant syndrome, supine hypertension, coronary artery disease, arrhythmia, chronic heart failure	Nausea, headache, increased blood pressure, hallucination, and anorexia
Ergometrine	An ergot alkaloid, which causes arterial vasoconstriction by stimulation of α-adrenergic and serotonin receptors as well as inhibiting the release of endothelial-derived relaxation factor	1 mg used in trials (OD) Licensed for 250–500 μg in postpartum haemorrhage	Eclampsia; first stage of labor; induction of labor; s stage of labor; sepsis; severe cardiac disease; severe hypertension; vascular disease	Abdominal pain; arrhythmias; chest pain; coronary vasospasm; dizziness; dyspnoea; headache; hypertension; myocardial infarction; nausea; palpitations; pulmonary edema; rash; tinnitus; vasoconstriction; and vomiting
Midodrine	A prodrug that is converted into desglymidodrine, which works on α_1_-adrenergic receptors to increase vascular tone	2.5–10 mg TDS	Aortic aneurysm; blood vessel spasm; bradycardia; cardiac conduction disturbances; cerebrovascular occlusion; congestive heart failure; hypertension; hyperthyroidism; myocardial infarction; narrow-angle glaucoma; proliferative diabetic retinopathy; serious prostate disorder; urinary retention	Chills; flushing; gastrointestinal discomfort; headache; nausea; paraesthesia; piloerection; scalp pruritus; supine hypertension (dose-dependent); and urinary disorders
Pyridostigmine	An anticholinesterase that reduces the breakdown of acetylcholine in the synaptic cleft	60 mg used in trials (30 mg BD/60 mg OD) 30–120 mg licensed for multiple sclerosis	Intestinal obstruction; urinary obstruction	Abdominal cramps; diarrhoea; excessive tearing; hypersalivation; nausea; and vomiting
Yohimbine	Presynaptic α_2_-adrenergic receptor antagonist, which may lead to increased norepinephrine release	2–5.4 mg (TDS) used in clinical trials	Pregnancy, hypersensitivity, children, and panic disorder	Anxiety; irritability; headache; sweating; nausea; tachycardia; priapism; worsening of panic attacks; vomiting; dizziness; nervousness; restlessness; reduced urination; hypertension; tremors; and skin flushing

The only medicine to receive marketing authorization for OH is midodrine in the United Kingdom, licensed for nOH. The National Institute for Health and Care Excellence (NICE) acknowledges the off-label use of fludrocortisone in OH^63^; however, there is no distinct guidance on the pharmacological management pathway of OH.

Pharmacotherapies used for OH can be divided into 2 broad categories: fluid expanders that increase the effective circulating blood volume and vasopressors that work to increase peripheral vascular resistance either by acting peripherally or centrally.

**Fludrocortisone** is a mineralocorticoid analogue (similar to aldosterone) that increases sodium and water retention (and potassium excretion), leading to an expanded intravascular volume and subsequent rise in BP. The evidence base for fludrocortisone is limited to several small studies, where it is commonly co-prescribed with other therapies^64,65^ and studied in small, heterogenous patient groups.^66^ Commonly used doses range between 0.1 and 0.2 mg/d. Fludrocortisone is slow-acting, taking ∼1–2 weeks to exhibit any benefit. It seems to work best in those with a low-fluid-volume status. The increased circulating volume can exacerbate cardiac and renal failure; the aldosterone-like action can cause hypokalemia and lead to significant worsening of hypertension, especially in SH and elderly patients.^19^

**Midodrine** is an oral prodrug, a peripheral selective α-1-adrenergic agonist, which acts as a vasoconstrictor, resulting in increased TPR. RCTs including double-blind placebo-controlled trials, supported by subsequent meta-analyses, identify midodrine as an effective treatment for OH.^67–71^ However, the presumptive use of midodrine in all cohorts with OH is unlikely to show benefit, for instance, in postoperative/acute OH patients.^72^ Midodrine has a relatively short half-life (approximately 4 hours), and the BP rises by approximately 10–30 mm Hg within 1–2 hours of intake of a 10-mg dose. The first dose is preferably given 30–60 minutes before the morning rise and daily dosage may vary greatly from 2.5 mg to 37.5 mg in desensitized patients. Doses can be individualized to a patient's activity and on an as-needed basis while avoiding doses after 6 pm to prevent SH. The side effects of midodrine include piloerection, scalp pruritus, and dysuria. SH and urinary retention are the most cited reasons for discontinuation. Midodrine is the only drug licensed for nOH in the United Kingdom. It was approved based on a reduction of the drop in 1-minute standing BP.

**Pyridostigmine** is a cholinesterase inhibitor that facilitates cholinergic neurotransmission at the level of the autonomic ganglia. It acts presynaptically in both sympathetic and parasympathetic ganglia to harness the patient's residual sympathetic activity. Hence, it has a more preferential increase in upright BP and is less likely to cause SH. The effects on BP rise are modest per se; approximately 4 mm Hg rise in standing SBP. Therefore, its use is probably limited to milder OH or as a combination therapy.^73,74^ Improvement in OH and associated symptoms with pyridostigmine is inferior to that with midodrine,^75^ fludrocortisone,^76^ and other experimental agents.^77^ Pyridostigmine 60 mg OD or 30 mg BD is used in these studies, a much lower dose than that used for myasthenia gravis (up to 120 mg). Side effects represent a hypercholinergic state including abdominal cramps, diarrhea, lacrimation, hypersalivation, nausea, and vomiting. Pyridostigmine is contraindicated in patients with urinary or intestinal obstruction.

**Atomoxetine** acts as a norepinephrine reuptake inhibitor by inhibiting the norepinephrine transporter (NET), thus increasing the concentration of norepinephrine in synaptic clefts. It uses residual sympathetic activity, which is responsible for the control of upright BP.^78^ It is currently licensed for attention-deficit hyperactivity disorder. A comparison of the efficacy of atomoxetine (10 or 18 mg) and midodrine in autonomic failure showed that atomoxetine produced a greater pressor response (mean difference between the treatment arms was 7.5 mm Hg; 95% CI, 0.6–15; *P* = 0.03). Atomoxetine but not midodrine improved OH-related symptoms compared with placebo.^79^ However, other studies have found that atomoxetine alone does not increase seated or standing BP but is effective when used in combination with α-2 antagonists such as yohimbine or with pyridostigmine.^78,80,81^ Significantly better results are noted in patients with MSA where post-ganglionic nerves are intact, with a higher resting norepinephrine level in contrast to the PAF and PD cohorts where resting norepinephrine levels are lower.^82^ Atomoxetine is contraindicated in pre-existing severe CV disease, potentially limiting its utility in elderly populations. The side effects include anxiety, depression, arrhythmias, and headaches; patients should be counselled on the potential for suicidal thoughts and behaviors.

**Droxidopa**, a norepinephrine prodrug, was approved by the FDA in 2014 for short-term use for symptomatic nOH.^19^ A meta-analysis of RCTs comparing droxidopa and midodrine demonstrated a mean increase of 6.2 mm Hg and 17 mm Hg in standing SBP, respectively, with a reduced relative risk of SH with droxidopa in comparison to midodrine.^83^ There has been no trial comparing midodrine and droxidopa directly, and as such, long-term efficacy remains unproven.^84–86^ Overall, most studies reported significant improvements in both standing BP and symptoms,^87,88^ and its outlook in treating short-term nOH looks promising.^89^ The doses used in the trials range from 100 mg to 600 mg 3 times daily while avoiding bedtime dosing to prevent SH. Some studies revealed a better response to droxidopa in patients with lower resting norepinephrine levels, indicating a degree of denervation supersensitivity. It is not available in the United Kingdom at the time of writing.

**Yohimbine**, an α-2 receptor antagonist, the pharmacological opposite of clonidine, increases sympathetic flow in the central and peripheral nerves by potentiating the release of norepinephrine. Previous studies showed its effectiveness in correcting PH related to tricyclic anti-depressants^90^; however, it was not effective in managing OH secondary to PD.^91^ At 5.4 mg oral dose 3 times a day, a strong protective effect against OH, surpassing the effect of pyridostigmine, was seen in patients with autonomic failure.^77^ It also had a synergistic effect when combined with atomoxetine in another study, suggesting that it can increase sympathetic reserve.^80^ However, it can lead to multiple adverse events including insomnia, sweating, suicidal ideation, and SH. The full benefits of this drug are still to be realized, and as such, the drug remains an investigational agent.

### Other Experimental Pharmacotherapies for OH

Various other therapies have been attempted in the past, with minimal degrees of success, although some newer therapies may hold promise. Erythropoietin, domperidone, and caffeine have shown efficacy in basic proof-of-concept studies^66,92–94^; however, they have not been tested in larger studies. Vitamin D and desmopressin have demonstrated comparatively weak results.^95,96^ More recently, potentially efficacious therapies have been more systematically validated. Ampreloxetine, a NET inhibitor like atomoxetine but with a longer half-life, was found to have a significant effect on BP in subjects with nOH in a phase II trial.^97^ Of note, the drug was granted Orphan Drug Designation status by the Food and Drug Administration (FDA)—specifically for patients suffering from nOH secondary to MSA.^98^ CERC-301 is an NMDA receptor antagonist and exhibits a protective effect against nOH, demonstrating safety in a phase 1 study.^99^ Rebreathing devices (NCT05908760) and continuous positive airway pressure (NCT05489575) are being considered in experimental studies for improvement in OH and SH, respectively.

### Therapeutic Options for Postprandial Hypotension

Management of PPH is rather challenging. Acarbose, previously used for type 2 DM, resulted in improved postprandial BP in comparison with placebo in a small study.^23^ It is postulated to act by delaying gut glucose absorption, resulting in reducing the breakdown of carbohydrates, which in turn has been shown to reduce PPH. Octreotide is a somatostatin analogue postulated to benefit PPH. It acts by inhibiting the release of gastrointestinal peptides, some of which have vasodilatory properties. A common starting dose is 25–50 µg, given subcutaneously 30 minutes before meal. Larger doses may, however, lead to cholelithiasis.^100,101^ Guar gum seems to work similarly to acarbose by reducing the magnitude of glucose absorption and delay in gastric emptying.^102^ Interestingly, based on a small study, caffeine (an adenosine antagonist) was noted to reduce PPH, possibly indicating that adenosine may contribute to inappropriate vasodilation.^88^

## PERSPECTIVES AND FUTURE CONSIDERATIONS

Overall, midodrine, fludrocortisone, and pyridostigmine are reasonable initial pharmacological options in the appropriate patients with OH. Atomoxetine seems to be gaining popularity with the obvious advantage of an available therapeutic repurposed option. Droxidopa is not yet approved or available worldwide. Ultimately, pharmacological therapies need to be tailored to underlying pathophysiology and individual patient needs. The proposed management algorithm is a practical and pragmatic framework for individualizing treatment for this debilitating condition (Figs. 3 and 4) and builds on previously published comprehensive reviews.^103,104^

Multifactorial ranging from heterogeneous disease populations, diagnostic challenges among nOH etiologies, and variable therapeutic endpoints used in clinical trials lead to building of robust evidence base.

This can be addressed by further building collaborative, cross-disciplinary clinical studies, and/or real-world evidence-building strategies such as international registries, where therapy is based on a personalized and stratified therapy approach that might lead to streamline management and care for these patients.

## CONCLUSIONS

OH is a prevalent condition affecting populations including the elderly and multi-morbid patients. The etiology of OH is variable, which makes management challenging. Multiple nonpharmacological and pharmacological therapeutic options are available, although the latter are mostly available as off-label for the indication of OH. The evidence base for these therapies is minimal; RCTs are either non-existent or unpowered, focusing on extremely specific subsets of the population, or with inconclusive results. Larger RCTs and/or well-designed observational studies may help identify factors that determine the success of a particular therapy. As such, management must be personalized, with a risk–benefit analysis of the condition itself and available therapeutic options, with the primary goal of ameliorating symptoms and improving quality of life.

## Supplementary Material
