# Supplementary material for: Treating Lows: Management of Orthostatic Hypotension
Source: J Cardiovasc Pharmacol. 2024 Sep 3;84(3):303–15. doi: 10.1097/FJC.0000000000001597 (PMC11368167; doi:10.1097/FJC.0000000000001597)
Supplement: Supplementary file 1 [file jcvp-84-303-s001.docx]

**Supplementary table: Evidence base of therapy for OH**

| Intervention type | Study ID | Study design | Subject characteristics | Results | Study limitations |
| --- | --- | --- | --- | --- | --- |
| Physical counter-pressure manoeuvre | Ten Harkel et al. 1994 [34] | Cross-over trial comparing leg muscle pumping (tiptoeing) and tensing (leg-crossing) | Healthy volunteers (aged 28-34) and patients with severe hypo-adrenergic OH (n=13) | In hypo-adrenergic patients, leg-crossing caused an increase in BP of 13mmHg (+/- 13); tiptoeing caused no significant difference in BP. | Open-label study design; potential for bias due no possibility of blinding |
|  | Tutaj et al. 2006 [35] | Cross-over trial comparing four physical counter-manoeuvres – bending forward, squatting, leg crossing and abdominal binding. | Patients with familial dysautonomia (n=17) | Squatting was the most effective manoeuvre, mean BP increased by 50.8mmHg (33.5 - 56; P = 0.002). BP was also increased significantly by bending forward (by 20.0 (17 - 28.5) mmHg; P = 0.005) and abdominal compression (by 5.8 (-1 - 34.7) mmHg; P = 0.04) - but not by leg crossing. | Errors in impedance cardiography due to movement artifacts  High subject dropout  Open-label study design |
|  | Van Lieshout et al. 1992 [36] | Cross-over trial comparing squatting and leg crossing | Healthy volunteers and patients with severe hypo adrenergic OH (n=13) | Leg crossing caused an increase in mean BP of 13mmHg (SD 6), while squatting increased mean BP by 44mmHg (SD 18). The BP increase in healthy volunteers was minimal, 4 mmHg for leg crossing and 12 for squatting.  Patients could stand for ≥ 10 mins with counter manoeuvres | Open-label study design, extremely small study |
|  | Van Dijk et al. 2006 [37] | Multicentre, prospective, randomized clinical trial comparing standard therapy vs standard therapy with training in physical counterpressure manoeuvres | Patients with vasovagal syncope and prodromal symptoms (n=223) | Syncope burden was significantly reduced for the trained group vs the controls (p = 0.004). | Open-label study design, long term efficacy unknown |
| Compression bandages | Podoleanu et al. 2006 [42] | Randomised cross-over study with and without elastic bandage of the legs and abdomen with tilt-table response as outcomes. Followed by 1-month open-label use of the leg elastic compression | Patients with symptomatic, progressive OH (non-nOH) (n=21) | Leg compression (40-60 mm Hg) reduced the drop in BP (approx. 20 mm Hg; p=0.003 vs control) during tilt test.  Patients experienced a reduction in OH related symptoms (measured by Specific Symptom Score questionnaire) after 1 month of compression stockings (p=0.01) | Concomitant medical therapy affecting orthostatic tolerance.  Open-label study design |
|  | Figueroa et al. 2015 [51] | Randomized crossover trial to compare elastic and drawstring abdominal binders with pressure adjustments on standing | Patients with nOH (n=13) | Abdominal binders with compression levels of 10 mm Hg caused the most blunting in the fall of SBP (conventional binder: mean rise 7mm Hg (p=0.03), Adjustable binder:11 mm Hg, (p=0.01) depending on the binder used)). | No information on sustained, long-term effects of intervention  Open-label study design |
|  | Fanciulli et al. 2016 [44] | Single-blinded placebo-controlled crossover design to evaluate elastic abdominal binders vs a placebo binder | Patients with PD and symptomatic OH (n=15) | Increased mean BP (10.2 mm Hg, Range:3.5-16.5, p=0.006) at 3-minute head up tilt test. After 4-week open-label usage, there was a significant reduction in symptoms according to OH questionnaire (p=0.003) | Single blind design for initial phase  Open-label study design for follow-up phase |
|  | Denq et al. 1997 [48] | Cross-over trial using head-up tilt testing to evaluate abdominal, leg or abdominal and leg compression, using an antigravity suit | Patients with nOH (MSA, PAF or autonomic neuropathy) (n=14) | Compression of abdominal and leg vascular beds was most efficacious, with improvement in both BP (approximate difference of means of 25mmHg, p<0.001) and subjective improvement in symptoms. Compression of the abdomen alone was also found to significantly improve both symptoms and standing BP (p<0.005) | Open-label study design  Lack of long-term assessment of clinical outcomes |
|  | Smit et al. 2004 [43] | Cross-over trial to test abdominal compression during a head up tilt test. | Patients with nOH (n=23) | Improvement of upright BP (by 30 mmHg (systolic) and 14 mmHg (diastolic), p <0.05)) and increased stroke volume (14% p<0.05). | Open-label study design |
|  | Yamamoto et al. 2006 [45] | Cohort study to assess post-haemodialysis (HD) supine and standing BP with and without the use of an abdominal binder | Patients on HD with post-dialytic orthostatic  hypotension (OH) (n=25) | SBP rose higher with the use of the abdominal band post haemodialysis (129.6+/-27.3 vs 117.2+/-22.6 mm Hg; P<0.05) | Open label study design  Effect of concomitant medications on intervention effects. |
|  | Protheroe et al. 2011 [46] | Randomized, cross-over, double-blind to compare graded calf compression stockings vs 2 placebo stockings | Healthy volunteers (n=15) | Calf compression did not significantly change cardiovascular or respiratory parameters during tilt test | Healthy subject study  Open label study design |
|  | Moein et al. 2017 [47] | Placebo controlled, double blind, cross-over RCT to trial a prototype portable calf active compression brace vs control | Healthy volunteers (n=14) | Calf compression significantly increased stroke volume (+5.20±2.34%, p = 0.05), but no significance for decreasing the HR (-5.12±2.41%, p = 0.06) or increasing SBP (+4.86±3.41%, p = 0.18) during tilt test. | Open-label study design  Lack of a control arm |
| Water bolus | Jordan et al. 1999 [54] | Cohort study measuring seated BP measurements across a 100-minute period post 480ml tap water bolus | Patients with severe OH due to autonomic failure (PAF, MSA) and controls (n=30) | Raised BP by 11mmHg (p<0.001) with peak 35 minutes after bolus | No dose-dependent relationship between intervention and effect found. |
|  | Shannon et al. 2002 [55] | Cohort study comparing standing BP pre- and post-drinking 480 mL of tap water at room temperature in less than 5 minutes | Patients with severe OH due to autonomic failure or orthostatic tachycardia due to idiopathic orthostatic intolerance (n=20) | Water drinking improved standing BP and orthostatic tolerance at 35 minutes (increase of BP of 31mmHg). But there remained a large orthostatic drop. | No dose-dependent relationship between intervention and effect found. |
|  | Young and Mathias 2004 [57] | Cohort study assessing supine and standing BPs were recorded pre- and post-ingestion of 480ml of distilled water. | Patients with autonomic failure (peripheral autonomic failure or MSA) (n=14) | Oral ingestion of water resulted in improvement in standing BP at 15- and 35-minutes post ingestion, (approx. 20mmHg improvement at 3minutes standing for both, p<0.001) as well as subjective reporting of OH symptoms | No dose-dependent relationship between intervention and effect found. |
|  | Senard et al. 1999 [50] | Cohort study whereby participants drank a 500ml water bolus and had a standing BP taken at 60 minutes | Patients with PD and resultant OH (n=13) | No significant change in supine SBP or DBP or in HR | No dose-dependent relationship between intervention and effect found |
| Water bolus, physical counter manoeuvres and compression bandaging | Newton and Frith 2018 [49] | Cross over RCT-open label trial, with the following interventions: Bolus water drinking (480 mL tap water consumed within 5 minutes), physical counter-manoeuvres (standing cross-legged), compression stockings (on the upper thigh [23–32 mm Hg]), and abdominal compression (elastic belt). | Patients over 60 years of age with OH (n=25) | Primary outcome (proportion of participants whose SBP drop improved ≥10 mm Hg), was achieved by water bolus in 56% and abdominal compression in 52%. Water boluses (480 ml of tap water consumed within 5 min) were the most efficacious. While calf compression was least efficacious with 32% responding | As control BP measurements were performed first and closer in time to meals, potential postprandial hypotension during control measurements could exaggerate the interventions’ effects. |
| Midodrine | Low et al. 1997 [63] | Multicentre, randomised placebo-controlled trial comparing placebo vs midodrine (10mg TDS) | Patients with nOH (n=171) | Midodrine (10mg) improved standing SBP (p<0.001) and reported symptoms (p=0.001). Patient and investigator global symptom relief scores also both improved (p=0.03 and p<0.001 respectively) | Patient dropouts due to noncompliance and confounded by taking other vasoactive medications,  Open-label study design  Lack of assessment of long-term effects, beyond 6 weeks |
|  | Wright et al. 1998 [64] | Double-blind, placebo-controlled, four-way crossover trial studying placebo vs midodrine 2.5, 10 or 20mg | Patients with nOH (n=25) | Midodrine increased standing BP with peak at 1-hour post ingestion. Improvement of symptoms was significantly higher for 10 and 20mg vs placebo. | Lack of long-term assessment of relevant clinical outcomes |
|  | Phillips et al. 2014 [67] | Randomised cross-over study assessing BP and symptoms on a tilt table with either midodrine (10mg) vs no medication | Patients with spinal cord injury (C4-T5) and equal number of controls, able bodied, age and sex matched individuals (n=10) | Midodrine administration led to a 59% improvement in orthostatic tolerance (p<0.01) | Lack of blinding and placebo control |
|  | Jans et al. 2015 [68] | Double-blind, randomized trial assessing midodrine hydrochloride (5mg) or placebo 1 hour before mobilization at 6 and 24 h post- operatively | Patients scheduled for total hip arthroplasty under spinal anaesthesia (n=120) | Use of midodrine did not significantly reduce the presence of OH post surgically | Patients with preoperative OH were not stratified or excluded. |
|  | Jankovic et al. 1993 [65] | Double-blind, placebo-controlled study, studying the efficacy of midodrine (2.5, 5 or 10mg TDS) | Patients with OH (n=97) | Midodrine (10 mg) increased standing SBP by 22mmHg (p<0.001) and provided some symptomatic relief (p<0.05) for dizziness, weakness, syncope, low energy, and impaired ability to stand. | Acute effects 1 hour post dose alone was studied |
| Pyridostigmine | Singer et al. 2003 [69] | Prospective open label single dose trial studying the effects of pyridostigmine (60mg) | Patients with nOH (MSA, PD, diabetic neuropathy, amyloid neuropathy, and idiopathic autonomic neuropathy) (n=15) | Non-significant increase in supine BP, but significant increase in orthostatic BP and reduction in BP fall during tilt test (p<0.05). | Acute effects (1 hour post dose) were studied. |
| Pyridostigmine vs Pyridostigmine and midodrine | Singer et al. 2006 [70] | Double-blind, randomized, 4-way cross-over study with the following treatment arms:  Pyridostigmine bromide (60mg OD), alone vs pyridostigmine bromide (60mg OD) with midodrine (2.5 mg) vs pyridostigmine bromide (60mg OD) with midodrine (5 mg) vs placebo. | Patients with nOH (MSA, PAF, autoimmune autonomic neuropathy, diabetic autonomic neuropathy, or otherwise unspecified nOH) (n=58) | Active treatment led to an improved standing DBP (p=0.02). Pairwise comparison showed that pyridostigmine alone or with 5mg of midodrine significantly reduced DBP falls (p = 0.04 and p = 0.002 respectively). The improvement in BP significantly regressed with an improvement in OH symptoms | Lack of longer assessment of clinical effects beyond 6 hours post-treatment |
|  | Byun et al. 2017 [71] | Randomized, open-label clinical trial with the following branches: Midodrine (2.5mg BD) vs pyridostigmine (30mg BD) vs combined Midodrine (2.5mg BD), and pyridostigmine (30mg BD) | Symptomatic nOH patients (n=87) | Orthostatic SBP and DBP drops improved significantly at 3 months for all treatment arms (p<0.01 and p<0.05 respectively). Symptom improvement was highest with midodrine (p<0.01) | Open-label study design |
| Pyridostigmine vs Yohimbine | Shibao et al. 2010 [73] | Randomised single blind placebo controlled cross-over study to assess the following medications:  Pyridostigmine (60mg), yohimbine (5.4 mg) and combined pyridostigmine (60mg), yohimbine (5.4 mg) vs placebo | Patients with severe autonomic failure (MSA, PAF, PD) (n=31) | Yohimbine alone increased DBP at 60 minutes post administration vs placebo (p<0.001), while pyridostigmine administration did not lead to a significant increase. Both drugs combined led to an increase in DBP (p = 0.006), but this was not significantly different from yohimbine alone (p=0.504). | Single-blind study design  Lack of longer assessment of clinical effects beyond 1-hour post-treatment |
| Fludrocortisone vs domperidone | Schoffer et al. 2007 [62] | Double‐blind randomized controlled crossover trial  assessing fludrocortisone (0.1 mg OD and placebo BD) or domperidone (10 mg TDS) | Patients with idiopathic PD with stable response to medications and OH symptoms (n=17) | Both medications had a significant improvement in the orthostatic domain of the Composite Autonomic Symptom Scale (COMPASS‐OD) (p = 0.04 for domperidone and p=0.02 for fludrocortisone), and the clinical global impression of change (CGI). There was a trend towards improvement in BP, greatest in domperidone, but neither drug reached statistical significance | High subject dropout rate  Lack of dose dependent response assessment (only single medication doses used) |
| Droxidopa | Hauser et al. 2014 [80] | Multicentre double blind RCT comparing.  Titrated droxidopa (100-600mg TDS) vs placebo | Parkinson’s patients with documented nOH (n=51) | No difference in primary outcome (change in Orthostatic Hypotension Questionnaire (OHQ)) (p = 0.98) | Potential for variability in treatment response due to dose optimization phase prior to maintenance period |
|  | Biaggioni et al. 2015 [81] | Open-label Randomised control trial to assess titrated droxidopa (100-600 mg, 3x daily) vs placebo | Patients with symptomatic nOH and PD, MSA, PAF, or nondiabetic autonomic neuropathy (n=101) | No difference in primary outcome: patient-reported scores on the OHQ and BP measurements.  Post-hoc analysis indicated droxidopa may improve symptoms (p=0.013) | Open-label design of initial phase  Lack of monitoring of the use of countermeasures in patients (i.e., compression garments)  Observed carry-over therapeutic effect of droxidopa in the placebo phase. |
|  | Hauser et al. 2015 [82] | Phase III, multicentre, double‐blind, placebo controlled randomised control, parallel‐group trial, comparing titrated droxidopa (100-600mg TDS) vs placebo | Patients with symptomatic nOH resulting from PD (n=171) | There was a significant reduction in item 1 ("dizziness, lightheadedness, feeling faint, or feeling like you might black out") of the Orthostatic Hypotension Symptom Assessment (OHSA) subsection of the OHQ at maintenance week 1 (p=0.018), but there was a loss of significance vs placebo from weeks 2-8 | Possible impact of concomitant medications that patients were allowed to take concurrently (i.e., midodrine, PD drugs, short-acting anti-hypertensives) |
|  | Kaufmann et al. 2014 [84] | Double-Blind, Placebo-Controlled, Crossover Study comparing patient specific L-DOPS doses in dose ranging study (200-2000mg) | Severe symptomatic OH patients (MSA or PAF) (n=19) | L-DOPS significantly raised supine and standing BP (p<0.001). Orthostatic tolerance was significantly increased at 3 minutes with L-DOPS with 94% able to stand vs 84% after having the placebo (p<0.001). | Lack of longer follow-up of clinical outcomes beyond 3 days |
|  | Kaufmann et al. 2014 [84] | Multicentre double blind, placebo controlled open-label RCT of droxidopa (100–600 mg TDS) vs placebo (in responders to droxidopa) | Patients with symptomatic nOH due to PD, MSA, PAF, or nondiabetic autonomic neuropathy (n=162) | Droxidopa was effective vs placebo at reducing OH symptoms (measured using OHQ composite score, p=0.003) and improving standing BP (p<0.001) at 7 days of use | Open-label study design |
| Atomoxetine | Shibao et al. 2007 [78] | Randomized, single-blind, placebo-controlled crossover study of atomoxetine (18mg) vs placebo | Patients with severe autonomic failure (central or peripheral autonomic failure) (n=21) | Atomoxetine significantly improved seated and standing BP in central autonomic failure patients (p = 0.004 and p=0.016 respectively), but not those with peripheral failure | Lack of longer assessment of clinical effects beyond 1-hour post drug administration |
| Atomoxetine vs midodrine | Ramirez et al. 2014 [74] | Randomized, single-blind, placebo-controlled crossover trial with the following regimen:  on separate days, patients received atomoxetine (18 mg), midodrine (5-10 mg), or placebo | Patients with severe autonomic failure (PAF, MSA, and PD) (n=65) | Both atomoxetine and midodrine increased upright BP compared to placebo, but atomoxetine had a relatively greater increase (p=0.03) and atomoxetine alone significantly improved OH-related symptoms compared to placebo (p=0.02) | A lack of accounting for BP and other relevant clinical outcomes beyond 1-hour post-drug administration |
|  | Byun et al. 2020 [75] | Open label randomised control trial comparing atomoxetine (18mg OD) vs midodrine (5mg BD) for 1 month | Patients with symptomatic nOH (n=50) | Improved orthostatic SBP and DBP for both groups at 1 month compared to baseline (p<0.01 for all). Atomoxetine alone showed improvement in symptoms at 1 month (p<0.05) | Open-label study design. |
| Atomoxetine vs yohimbine vs combined | Okamoto et al. 2012 [76] | Single blind, cross over, placebo-controlled trial with the following branches:  Yohimbine (5.4mg), atomoxetine (18mg), or combined yohimbine (5.4mg) and atomoxetine (18mg) vs placebo | Patients with severe peripheral autonomic failure (n=17) | Only combined treatment caused an improvement in seated SBP (p<0.001), and improvement in orthostatic symptoms (p=0.013) | Plasma atomoxetine and yohimbine levels were not measured. Both are metabolized via CYP450 and have the potential for drug-drug interactions in vivo.  Lack of longer assessment beyond 1-hour post-administration |
| Atomoxetine vs Pyridostigmine vs combined | Okamoto et al. 2019 [77] | Single blind, placebo controlled cross-over study with:  Placebo (OD), atomoxetine (18mg OD), pyridostigmine bromide (60mg OD), or combined atomoxetine (18mg OD), pyridostigmine bromide (60mg OD) | Patients with nOH and severe autonomic failure (n=12) | Improvement in seated BP, orthostatic symptoms only in the combined arm (p<0.001) | Failure to assess outcomes beyond 1-hour post-treatment. |

Abbreviations:

OH – orthostatic hypotension, nOH – neurogenic orthostatic hypotension, BP – blood pressure, HR – heart rate, SBP – systolic blood pressure, DBP – diastolic blood pressure, PD – Parkinson’s disease, PAF – pure autonomic failure, MSA – multiple system atrophy, OD-once daily, OHSA- Orthostatic Hypotension Symptom Assessment, OHQ- Orthostatic Hypotension Questionnaire
